# The Application of Biologic and Synthetic Bone Grafts in Scoliosis Surgery: A Scoping Review of Emerging Technologies

**DOI:** 10.3390/healthcare13182359

**Published:** 2025-09-19

**Authors:** Nikolaos Trygonis, Ioannis I. Daskalakis, Christos Tsagkaris

**Affiliations:** 1 Klinik für Orthopädie, Hand- und Unfallchirurgie, Stadtspital Zürich Triemli, 8063 Zurich, Switzerland; 2 Department of Orthopaedic Surgery, University Hospital of Heraklion, 71110 Heraklion, Greece; 3 Faculty of Medicine, Aristotle University of Thessaloniki, 54124 Thessaloniki, Greece

**Keywords:** scoliosis surgery, bone grafts, autograft, synthetic grafts, 3D printing, bone defects, biomaterials

## Abstract

**Background**: Spinal deformity correction surgery, particularly in scoliosis, often necessitates long fusion constructs and complex osteotomies that create significant structural bone defects. These defects threaten the integrity of spinal fusion, potentially compromising surgical outcomes. Bone grafting remains the cornerstone of addressing these defects, traditionally relying on autologous bone. However, limitations such as donor site morbidity and insufficient graft volume have made urgent the development and adoption of biologic substitutes and synthetic alternatives. Additionally, innovations in three-dimensional (3D) printing offer emerging solutions for graft customization and improved osseointegration. **Objective**: This scoping review maps the evidence of the effectiveness of the use of biologic and synthetic bone grafts in scoliosis surgery. It focusses on the role of novel technologies, particularly osteobiologics in combination with 3D-printed scaffolds, in enhancing graft performance and surgical outcomes. **Methods**: A comprehensive literature search was conducted using PubMed, Scopus, and the Cochrane Library to identify studies published within the last 15 years. Inclusion criteria focused on clinical and preclinical research involving biologic grafts (e.g., allografts, demineralized bone matrix-DBM, bone morphogenetic proteins-BMPs), synthetic substitutes (e.g., ceramics, polymers), and 3D-printed grafts in the context of scoliosis surgery. Data were extracted on graft type, clinical application, outcome measures, and complications. The review followed PRISMA-ScR guidelines and employed the Arksey and O’Malley methodological framework. **Results**: The included studies revealed diverse grafting strategies across pediatric and adult populations, with varying degrees of fusion success, incorporation rates, and complication profiles. It also included some anime studies. Emerging 3D technologies demonstrated promising preliminary results but require further validation. **Conclusions**: Osteobiologic and synthetic bone grafts, including those enhanced with 3D technologies, represent a growing area of interest in scoliosis surgery. Despite promising outcomes, more high-quality comparative clinical studies are needed to guide clinical decision-making and standardize practice.

## 1. Introduction

Scoliosis is estimated to affect approximately 2–3% of individuals globally, with adolescent Idiopathic Scoliosis (AIS) representing the most prevalent form. Approximately 0.7% of patients with AIS progress to the point where surgical correction of the deformity becomes necessary, often in response to functional or aesthetic limitations [1]. Salvage surgery is most often performed with spinal fixation and long-segment fusion, frequently combined with column resections and/or osteotomies to restore global spinal balance. The main osteotomies described include Smith–Petersen osteotomy (SPO), pedicle subtraction osteotomy (PSO), bone–disc–bone osteotomy (BDBO), and vertebral column resection (VCR), in order of increasing complexity [2]. These procedures carry a risk of bone defects and pseudarthrosis, with rates ranging from 2% in AIS [3], to 2.2% in neuromuscular scoliosis [4], and up to 6.3% in adult deformities [4]. Such complications remain a major challenge, particularly in multi-level fusions or malpositioned grafts.

Moe first used autologous iliac crest bone graft (ICBG) for spinal fusion in 1958 [5]. ICBG remains the gold standard due to its biologic activity and fusion potential. However, its use is limited by donor-site morbidity, including infection (1.40%), seroma (0.64%), hematoma (1.49%), and iliac spine fracture (0.16%), often requiring reoperation [6]. These limitations, alongside technological advances, have driven the development of alternatives such as demineralized bone matrix (DBM), osteobiologics, bioactive peptide scaffolds, and ceramic bone substitutes [7]. Each material has distinct osteogenic, osteoconductive, and osteoinductive properties, with specific limitations.

Ceramic substitutes such as hydroxyapatite (HA) and tricalcium phosphate (TCP) share chemical similarities with bone. Although not osteogenic, they offer biocompatibility, osteoconductivity, and favorable mechanical properties [8]. Demineralized bone matrix (DBM), composed of collagen, non-collagenous proteins, and growth factors, provides both osteoconductive and osteoinductive effects and is available in various forms, including putty, facilitating application to defect sites [9].

More recently, three-dimensional (3D) printing has enabled patient-specific scaffolds with improved osteointegration and mechanical fit [10]. While promising, these technologies lack robust longitudinal and comparative studies, limiting clinical adoption.

Despite numerous available grafts, the expanding landscape of biomaterials and technologies has created uncertainty regarding optimal choice. A pragmatic review of current evidence will assist in clarifying effectiveness, benefits, and limitations of grafting options in scoliosis surgery. It will also provide an insight in the direction of ongoing trials.

The aim of this scoping review is to provide a comprehensive overview of biologic and synthetic bone grafts in scoliosis surgery, focusing on: (1) available materials, devices, and implants; (2) reported outcomes and complications; and (3) knowledge gaps and future research directions. This review seeks to guide surgical decision-making and inform future innovation in the management of scoliosis-related bone defects.

## 2. Materials and Methods

To conduct this review, a structured literature search was conducted across PubMed, Scopus, and the Cochrane Library seeking to identify observational studies, reviews, and clinical trials using biologics, biomaterials, and 3D technology in spine deformity surgery. To qualify for inclusion, studies had to focus on scoliosis surgery with spinal fusion using a graft material. The included studies had to report on clinical or radiological outcomes of the graft material used.

The search string using Boolean operators (AND, OR) included the following combinations: (scoliosis surgery OR spine deformity surgery) AND (bone defects OR pseudarthrosis) AND (bone grafts OR osteobiologics OR synthetic grafts OR ceramics OR 3D-printed scaffolds OR bone substitute).

Studies were restricted to those published from 2009 to 2025 to capture either emerging devices or devices that have been established in the last decade the use of which has been promptly documented in scientific discourse. We also included ongoing clinical trials researching biomaterials for spinal deformity surgery. Only English-language articles with available full text were chosen. All records identified were exported to the reference management program Zotero 6.0 to remove duplicates before screening. This scoping review was not registered at PROSPERO.

Study selection was conducted in two steps: first, titles and abstracts were screened, followed by full-text analysis of potentially relevant articles. Each study was extracted according to the following information: author(s), year of publication, country of origin, type of study (clinical trial, cohort, case series, preclinical), type of bone graft (autograft, allograft, DBM, synthetic ceramic, BMP, or 3D-printed material), patient population and age group, surgery performed, outcome variables (e.g., fusion rate, graft incorporation, complications), length of follow-up, and any reported limitations or complications.

**Figure 1:**
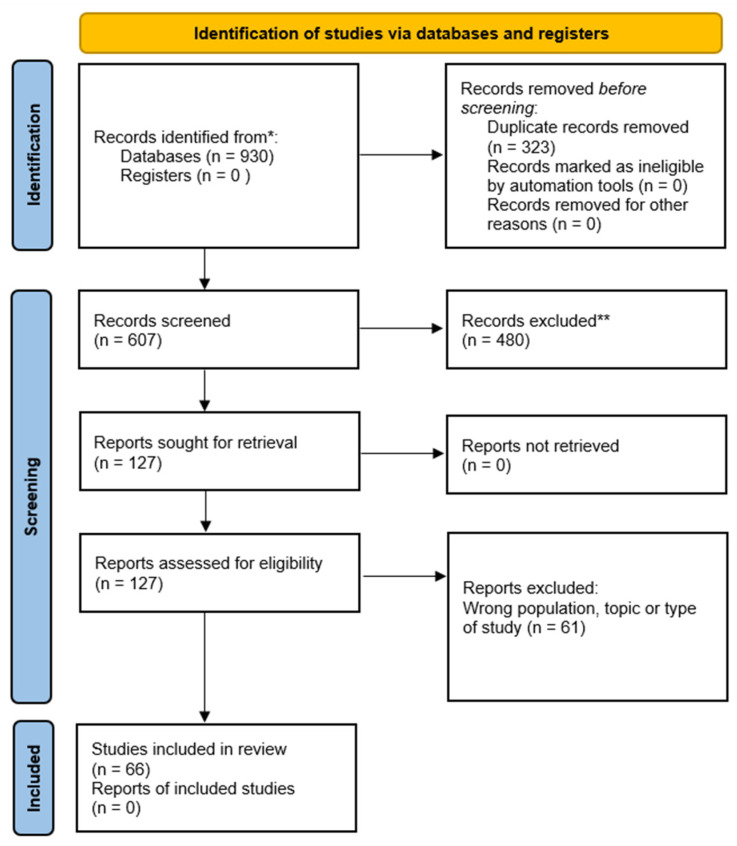
Literature search and identification flowchart as per PRISMA guidelines. * Refers to Databases used PubMed, Scopus, and the Cochrane Library. ** Refers to reasons for study exclusion: no referring to spinal deformity surgery, not using grafts, not reporting outcomes, not available in English.

The study selection process was documented by means of a PRISMA-ScR flow diagram (Figure 1).

Trends were summarized, where applicable, using tables with consideration of the distribution of studies and associations between graft type and clinical outcome. Descriptive synthesis also identified research gaps, particularly regarding long-term outcomes, cost-effectiveness, and the comparative efficacy of biologic versus synthetic graft materials in scoliosis surgery.

Bias assessment of clinical studies has been performed by means of the JBI Assessment toolkit and its specialized matrix for appraising case series.

## 3. Results

### 3.1. Overview of Included Studies

A systematic search of four electronic databases initially identified 930 records. After removing 323 duplicates, 607 records remained and were screened by title and abstract. Of these, 127 full-text articles were assessed for eligibility, and 66 met the inclusion criteria and were included in this review. Among the included studies, 21 were retrospective cohorts or randomized controlled trials (RCTs) and are presented in Table 1. In addition, two preclinical (non-human) studies were identified. The remaining studies were used for background/bibliographic purposes. Patient populations included adolescents and adults undergoing scoliosis correction surgery for idiopathic, congenital, or neuromuscular etiologies. The most frequently reported surgical treatments were posterior spinal fusion (PSF), vertebral column resection (VCR), and pedicle subtraction osteotomy (PSO).

An extensive range of the materials used in the form of bone grafts was discussed, with emphasis put on seven primary subclasses, autografts, allografts, and demineralized bone matrix (DBM), ceramics (hydroxyapatite [HA], beta-tricalcium phosphate [beta-TCP]), bioactive glass, bone morphogenetic proteins (BMPs), bioactive peptides, and 3D-printed scaffolds. Some of the outcomes measured in these studies were fusion rates, time needed to incorporate the graft, profile of complications, radiographic and histological evidence of healing, and also in some studies cost-effectiveness.

### 3.2. Types of Graft and Major Conclusions

The 23 studies that were incorporated were categorized according to the kind of bone graft used as a variable in the study of scoliosis surgery. The five categories of grafts were classified as: (1) autografts, (2) allografts, demineralized bone matrix (DBM), (3) ceramic-based grafts (e.g., HA, 8-TCP) and bioactive glass, (4) bone morphogenetic proteins-BMPs and bioactive peptides, (5) and even the 3D-printed scaffold emergent. There are results for each category using data from the table as well from other references/reviews. An overview of available grafts is presented in Table 2.

#### 3.2.1. Biologic Bone Grafts—Autografts

Autologous bone grafts serve as the gold standard in spinal fusion due to their inherent osteogenic, osteoinductive, and osteoconductive properties. Clinical attention has begun to shift from ICBG to local bone grafts, which are harvested intra-operatively from the spinous processes, laminae, and facet during exposure in posterior surgery [15]. Crostelli et al. reported 100% “solid fusion” in 108 females without extenders at 1.3 years and no implant failure [34], and Yataganbaba et al. found 99.1% fusion in 218 AIS patients using local bone graft (LBG), introducing a pragmatic “bone mass per segment” metric, with pseudarthrosis < 1% [14]. However, both reports—despite impressive figures—are limited by relatively short follow-up, potential selection bias, and variable definitions/imaging thresholds for “solid fusion”, which may inflate success rates.

Comparative data remain mixed but quite reassuring. Yang et al. [35] compared the outcome of ICBG with that of LBG in a cohort of 50 AIS patients, showing both groups maintained their clinical fusion results at 5 years. And while the ICBG group had slightly less radiographic correction loss as well (4.5° vs. 8.5°), there was no significant difference for patient-reported SRS-22 scores. The small cohort raises concerns about statistical power for patient-reported outcomes, and the clinical significance of a few degrees’ difference is debatable. Systematic comparisons cite 95% fusion with LBG, yet these syntheses aggregate heterogenous studies. One major deficiency of LBG, particularly in long segment fusions, is that the volume of graft material is limited. Giorgi et al. showed that pairing LBG with collagen–hydroxyapatite extenders (e.g., RegenOss) can meet volume needs without sacrificing fusion [24]. While biologically autograft remains “supreme” for its viable cells and native cues [15], real-world strategies increasingly adopt hybrid constructs—LBG augmented by synthetic scaffolds or osteobiologics—to balance biology with volume and reduce donor-site morbidity; Buser et al. review such combinations as achieving high fusion rates [36]. Still, most reports are retrospectively observational, with short- to mid-term horizons and diverse adjuncts, leaving unanswered questions about cost-effectiveness, infection risk, and long-term hardware survival across graft choices.

#### 3.2.2. Biologic Bone Grafts—Allografts

Allografts are an important alternative for treatment of bone defects in spinal fusion, especially when there is inadequate autograft volume. The most common types are freeze-cancellous allograft, DBM, and structural cortical allograft. It has been shown that allografts can achieve fusion rates on par with autograft with or without the use of autograft itself [16,17,19,37,38]. However, these equivalence claims largely stem from heterogeneous, mostly retrospective cohorts with variable imaging criteria and follow-up durations; apparent parity may be sensitive to stricter CT-based definitions and longer-term horizons. There was no difference between MISS (minimal invasive) versus COSS (conventional open) in a retrospective study of 86 AIS patients from Yang et al. [15] between different subgroups (allograft vs. DBM vs. demineralized cancellous bone chips).

Cortical strut allografts (i.e., fibular or femoral segments) have been employed for mechanical support and anterior column reconstruction. While infrequently described in the AIS, their efficacy has been demonstrated in severe kyphoscoliosis and revision deformity operations, stabilizing pedicle screws during fusion while remodeling over time [17]. Sinagra et al. assessed whether the quantity of allograft per level fused and the inclusion of autograft influenced the fusion rate in T1–T11 implantations for idiopathic adolescent scoliosis (mean 16 years). Subjects received 10 or 15 g of allograft per level of fusion. They found no difference in terms of allograft cm level increase or being mixed with autograft (*p* = 0.3258) [19]. A simple gram-per-level metric may be too crude; it ignores fusion surface area, construct length, and local biology, so the null finding should be interpreted cautiously and may not generalize to longer thoracolumbar or pelvic constructs.

The current trend is to incorporate osteobiologics with allografts to maximize filling of bone defects and to promote new bone formation. Onafowokan et al. compared BMP-2 vs. traditional (i.e., non-BMP) usage of allograft/DBM/autogenous marrow aspirate and found no difference in the rate of fusion [39]. In addition, in a novel IBM (integrative bone matrix) described by Passias, 20 patients were retrospectively examined at 3-, 6- and 12-month intervals after spinal fusion surgery receiving a novel IBM as bone graft. The patients showed a 100% fusion rate at 12 months with no graft-associated adverse events [31]. However, the series is small, uncontrolled, and short-term; 12-month “fusion” may reflect imaging thresholds rather than durable arthrodesis or hardware survival.

#### 3.2.3. Synthetic Bone Grafts

Synthetic materials are biodegradable bone graft substitutes usually used in combination with autogenous bone or bone marrow aspirate (BMA). Among the primary synthetic materials used are calcium phosphate-based ceramics, HA, β-TCP, and bioactive glass. These synthetics primarily act as osteoconductive scaffolds which are mechanically stable in low-load conditions. β-TCP has higher porosity and resorption capacity compared to HA, facilitating faster remodeling but potentially requiring mechanical support when used alone. Composite ceramics, combining β-TCP with HA, enhance graft longevity and structural integrity [40]. Critically, most “success” with synthetics reflects use as extenders alongside autograft/BMA rather than as true stand-alone substitutes.

In scoliosis correction procedures, β-TCP and HA blocks have shown promising outcomes. Giorgi et al. reported that HA-Collagen blocks used in adult spinal deformity cases fused well in 95% of patients at a 36-month follow-up [24]. Similarly, Lerner et al. [27] reported good results with the use of β-TCP as a bone extender in posterior fusion for adult idiopathic scoliosis. They reported no significant differences between the two groups and the overall loss of correction was 2.6 degrees for the β-TCP group and 4.2 degrees for the ICBG group. These findings are encouraging but limited by retrospective design and mixed populations (adult and adolescent spine deformity).

Bioactive glass, particularly 45S5 BAG, has gained attention due to its unique ability to form a hydroxycarbonate apatite (HCA) layer, promoting osteoblast attachment and gene expression favorable to bone formation. Studies in adolescent idiopathic scoliosis (AIS) have demonstrated that BAG achieves fusion rates comparable to iliac crest autografts without donor site morbidity. A prospective pediatric cohort study found BAG putty and granules to be safe and effective in posterior fusion for both idiopathic and non-idiopathic scoliosis, maintaining Cobb correction at 24 months and demonstrating no device migration or rod issues [30]. Recent meta-analysis data reinforce these findings: when mixed with local autograft, BAG fusion rates (~89.6%) approximate those of autograft alone (~91.6%), though standalone BAG fusion is far lower (~33.6%) [41]. Newer mesoporous BAG doped with therapeutic ions like strontium or copper show enhanced osteogenic signaling in preclinical models, though clinical translation in deformity surgery remains pending [42,43].

Another promising synthetic is silicate-substituted calcium phosphate (Si-CaP). In scoliosis surgery specifically, Lerner and Liljenqvist et al. demonstrated the safe use of Si-CaP combined with bone marrow aspirate (BMA), achieving solid fusion with no significant complications [25]. Because Si-CaP was paired with BMA, the independent contribution of the ceramic is unclear; sample sizes are small and head-to-head randomized comparisons with autograft or other extenders are lacking.

Generally, ceramics—with their customizable properties—function as both a workhorse and a messenger in delivering active molecules and other types of grafts to fill bone defects and achieve fusion. Practically, they help address graft volume constraints and avoid donor-site morbidity, but current evidence favors their role as adjuncts to local autograft/BMA or allografts.

#### 3.2.4. Bone Morphogenetic Proteins [BMPs]—Bioactive Peptides

Bone morphogenetic Proteins, especially rhBMP-2, have osteoinductive abilities. Experience is another weapon in our arsenal for bone defects. A large retrospective study (2658 AIS cases), showed BMP use reduced pseudarthrosis reoperations (<1 vs. 18.4% in non-BMP long fusions) [44]. But rhBMP-2 also has a high rate of heterotopic ossification and inflammation that depends on dosage. For example, a blistering skin reaction can occur at the infected wound bed after exposure to rhBMP-2 over time. This is why its pediatric use in scoliosis is limited only to cases where bone defects are anticipated to be high [45]. Approximately 40% of adult spinal deformity surgery cases now use BMP-2, a proportion that has steadily increased in recent years. Such cases often occur in older patients and may involve extensive circumferential fusions; costs for this group are higher [46]. So rhBMP-2 is used effectively in scoliosis revision cases, but at the same time one must carefully balance the potential for complications with its effectiveness.

Synthetic bioactive peptides like P-15 work as collagen-binding mimics to promote osteoblast activity. These are integrated into graft carriers such as anorganic bone mineral (i-Factor). One clinical trial showed P-15 + ABM matches or outperforms fusion results compared to local bone graft in adult spine cases, with no graft complications over 12 months [28]. These results are encouraging but derive from short-term endpoints and adult, largely non-deformity indications; durability beyond 12 months and generalizability to multi-level deformity constructs are uncertain. Another peptide is B2A, which is normally put on the surface of ceramic granules. In a multicenter, blinded pilot study on single-level lumbar fusions, patients receiving 750 µg/cm^3^ B2A-coated grafts achieved 100% fusion at 12 months compared to 78% with iliac crest autografts and had no increase in adverse events [29]. As a pilot, the study is small and not powered for safety. While no large randomized trials on P-15 alone have been conducted for scoliosis, several studies involving patients with adolescent idiopathic scoliosis (AIS) report satisfactory union without donor-site morbidity or graft-related adverse events when P-15 is used this way combined with anorganic bone mineral (ABM) carriers [28]. There are currently few direct studies on their use in scoliosis. Yet, because of the excellent results they demonstrate in other spinal surgeries, they are also recommended as an essential supplement for doing scoliosis bone defect resurfacing. Pragmatically, peptides (P-15, B2A) are best considered adjuncts/extenders in scoliosis until larger, scoliosis-specific randomized trials with standardized CT-based fusion criteria are conducted.

#### 3.2.5. 3D-Printed Guides and Scaffolds

The integration of 3D-printing technology into graft development has revolutionized the treatment of bone defects in spine deformity surgery, allowing for individualized active implants that treat structural defects and aid in mechanical stability [47,48]. Recently, Pesante et al. [49] has reported on the successful use of multilevel patient-specific 3D printed interbody cages to treat congenital scoliosis in juveniles with one-year follow-up results showing good fusion and no need for revision.

Now comes a new era for bone defect grafts in experimental research. We have developed a methacrylated hyaluronic acid/cobalt-doped ceramics splash-pattern “biocompatible ink” and achieved unparalleled slope of grafting material in bone defects [50,51,52]. With extrusion-based 3D printing, Lee et al. [53] also made use of the technique of 3D printing; they synthesized a novel bone substitute Alen-CDs@CDHA, which incorporates biphenyl diester-modified carbon dots (Alen-CDs) into 3D printed calcium-deficient hydroxyapatite scaffolds (CDHA). This material both had anti-osteoclastogenesis activity and fluoroscopically tracked the process of incorporation. Anti-osteoclastogenesis may aid early stability but could also slow remodeling; net effects on long-term fusion strength in load-bearing multi-level constructs are unknown. Another group, Saranti et al. [54], successfully prepared a scaffold of 3D-printed material with bioglass and carbon dots inside to promote bone growth and let medicine see where healing was going to take place. By incorporating nanotechnology into both bone produces and 3D printed scaffolds we are entering an era from which there’s no return: we can see fracturing live on screen and look at its result [55]. This vision remains speculative; real-time, in vivo, clinically actionable monitoring has not been validated in humans, and any “live” tracking must demonstrate that it changes decisions and outcomes without undue risk or cost. All studies to date are preclinical or still in animal models and have not yet entered clinical practice.

### 3.3. Bias Assessment

Twenty-one clinical studies have been assessed for bias. Differences were detected in terms of patients’ inclusion, either in a consecutive or in a complete manner. Nevertheless, all studies appeared to use sufficiently valid tools and methodologies for the evaluation and reporting of demographics and outcomes among included subjects (Table 3).

## 4. Discussion

Autologous bone grafts have been the mainstay for bone defects over decades, but their limitations are well known. Either ICBG or LBG get nearly 95% of fusions but currently long-segment fusions as in our operation start to limit their effectiveness [56]. After a volume threshold of less than 12 mL, the back construct in an artificial fusion enters failure mode [57]. This threshold likely reflects model-specific assumptions and may not generalize across construct lengths or patient biology, but it underscores the real-world volume constraint. These grafts fall under the limitations of patients, such as their age and bone quality. Thus, a need for biologic substitutes or novel osteobiologics to achieve reliable fusion with reduced morbidity is evident. From the data available in their review of the literature, Yoo et al. [58] pointed out that a combination of rhBMP-2 or DBM with autografts is able to bypass the volume limit, without causing rate fusion to decrease. Being used alongside autograft in patients with pediatric scoliosis, one series reported fusion rates as high as 99%, in comparison to the ~94% using autograft only [59]. However, much of this evidence is retrospective, with heterogeneous constructs and follow-up.

Allografts are finished-off products with moderate transformation as well as some induction. However, lack of an inherent osteogenic effect makes integration of third-generation products and their potential slow resorption an increasingly urgent issue in long-segment scoliosis constructs [60]. The data available from this period involving only allografts is out of date and compromised, as with large numbers of patients it is derived from retrospective observational studies lacking controls. At this stage they are probably best used in scoliosis surgery as substitutes or adjuncts to autologous grafts and osteobiologic, rather than stand-alone options [61].

Synthetics constitute one of the great advances in spine fusion surgery, leading to results equaling those of autograft. Ceramics such as those highlighted by Antonacci et al. [62] can provide great skeleton-like frame and transformation, but their brittleness and tensile strength are drawbacks. Hence, they have limited applicability to long-segment scoliosis surgery. Moreover, most favorable results derive from use as extenders with autograft/BMA rather than stand-alone substitutes, and cohort sizes are modest. Bioactive glass has also given rise to high hopes in a series of 84% fusion rates for pediatric scoliosis. There is currently no long-term, high-quality, multicenter data specifically adjusted to AIS patients [63], hence its current use as a means of avoiding autograft-donor complications.

BMPs yield similar rates of fusion to autograft and so accelerate the process of union where large bone defects are concerned, as Malham et al. [64] documented in their narrative review. Nonetheless their high risks and complications in soft tissue have left them with limited use, requiring very strict dosage protocols—like a powerful chemical agent held to keep high-risk scoliosis patients in check [65]. Use in pediatrics and deformity should therefore remain selective with a clear risk–benefit discussion.

Emerging evidence suggests that 3D printing technology may revolutionize the treatment of bone defects in scoliosis surgery. It provides more accurate fixation and 3D- printed biomodels with improved osteointegration and mechanical performance, but so far lacks long-term randomized controlled clinical trials available for scoliosis. All our research until now on custom-printed scaffolds has been preclinical work, which provides a foundation for future developments in 3D bioprinting. There are custom-printed raised patterns of allografts or ceramics that allow for individualized grafted replacement, which are of particular value in configurations that are often found in multi-level scoliosis reconstruction where irregular defects occur [66]. First-in-human studies with standardized imaging, growth considerations in pediatrics, and cost-effectiveness will be essential before routine adoption. The grafts described in this review are summarized on Table 2.

Emerging technologies are reshaping bone grafting in scoliosis surgery and are in preclinical studies.

Smart grafts equipped with sensors and reactive materials promise real-time monitoring of bone healing. Although these innovations are not scoliosis-specific, they can aid surgeons in achieving the optimal combination of available bone grafts, ensuring a successful fusion [67].

Another advance is 4D printing, introducing the ability of the material to respond to external stimuli. Imagine a material that can adapt its shape and stiffness in vivo, accommodating to changes to the microenvironment and mechanical stimuli to the changes in spinal alignment. Early studies suggest that 4D scaffolds could offer dynamic support in spinal fusion by adjusting to the biomechanical environment during healing, potentially reducing implant failure and improving fusion quality [68]. These concepts remain experimental; rigorous, scoliosis-specific trials with long-term follow-up, validated endpoints, and safety testing will be required before they can influence standard practice.

## 5. Limitations, Knowledge Gaps and Future Research

Despite the volume of literature on bone grafts in scoliosis surgery, significant gaps persist. Notably, pediatric versus adult subgroup analyses remain underdeveloped. As these populations, possibly due to different biological conditions and level of development and quality of bone, have different potential in growth, and because adult studies results are not the same as in minors, applying adult studies to the clinical practice of children (and vice versa) is not always correct. Studies reporting patient experience and functionality have also been scarce. This narrows decision making down to the understanding of biomechanics and objective clinical and radiological outcomes. Nevertheless, outcomes related to functionality and appearance need to be taken into account across the entire patient population and particularly among children and adolescents who receive surgery at a decisive period of their physical and mental development. Cost analyses comparing utility as well as ecological footprint across different materials and implants are necessary for ensuring financially and environmentally sustainable manufacturing and use of emerging technologies [69].

Additionally, a number of limitations of the present study need to be acknowledged. Searching literature in English might have led to excluding relevant studies published (partially or completely) in different languages. Moreover, reporting focused on mapping implants rather than exhaustively assessing or comparing their outcomes. A bias assessment has been conducted post-hoc in the frame of peer-reviewed assessment. The literature remains inconclusive in terms of the necessity of such an assessment in the context of a scoping review that rather intends to showcase the breadth of existing literature rather than providing an authoritative answer to a clinical dilemma. Therefore, it needs to be stated that studies have been assessed in manner enabling the authors to ensure wide reporting of evidence.

## 6. Conclusions

This scoping review underscores that managing bone defects during scoliosis surgery remains a central challenge to achieving optimal fusion outcomes. The graft landscape has evolved significantly, offering surgeons an expanded toolkit of biologic, synthetic, and technologically enhanced options. Success rests on hybrid, extender-based strategies highlighting the need for synthetic thinking on the surgeon’s side. Autograft remains the biologic benchmark but is volume-limited in long constructs; allograft and synthetics help bridge this gap, albeit with variable biologic potency and mostly short- to mid-term evidence. Potent osteoinductives (e.g., rhBMP-2) can reduce reoperation in selected high-risk scenarios but demand careful dosing, placement, and cost–risk counseling. Early 3D printing and “smart” scaffold technologies are promising yet remain in the preclinical/early clinical phase of trials and are not ready for routine use.

Challenges to be addressed include but are not limited to: (1) standardization of outcome reporting, (2) cost-effectiveness analyses, and (3) longitudinal studies and use of registers. The multitude of materials and devices as well as the involvement of multiple stakeholders—clinicians, manufacturers, and patients—makes precise, personalized, and human-centered used of implants urgent in order to attain favorable and long-lasting outcomes.

## Figures and Tables

**Table 1:** List of analyzed Studies.

Author(s) & Year [Citation]	Graft Type	Population	Surgical Procedure	Outcome Measures	Follow-Up	Key Findings-Limitations
Crawford et al. (2013) [11]	ICBG autograft	AIS	Posterior spinal fusion	Fusion rate, complications, OR time	24 month	94% fusion; low infection; increased blood loss Retrospective study 342 ICBG vs. 563 non-ICBG group
Erdem et al. (2025) [12]	Allograft vs. autograft	Adolescent scoliosis	Long-segment posterior fusion	Graft incorporation, loss of correction	45 month	100% fusion in both groups, retrospective study 54 patients 2 groups
Ohtori et al. (2016) [13]	ICBG	Degenerated lumbar kyphoscoliosis	Lateral interbody fusion	Blood loss, OR time, fusion rate	12 month	Good fusion; longer operation, prospective case series 12 patients
Yataganbaba et al. (2021) [14]	Local bone autograft	AIS	Posterior fusion	Radiologic fusion, pseudarthrosis	24.7 month	>99% fusion; 0.9% pseudarthrosis, retrospective review 218 patients
Yang et al. (2023) [15]	Allograft vs. DBM vs. demineralized cancellous bone chips	AIS	MIS vs. Conventional posterior fusion	Facet fusion by graft type	24 month	≥85% fusion across graft subgroups, prospective study 86 patients
Lansford et al. (2013) [16]	Freeze-dried allograft vs. allograft + Autograft	Adult deformity	Long-segment fusion	Fusion radiology, density	48 month	Effective fusion; slower graft resorption, retrospective comparative case series 26 patients with freeze-dried allograft vs. 21 with allograft + autograft
Bui et al. (2014) [17]	Structural allograft + local allograft	Neuromuscular scoliosis	Posterior fusion	Graft integration	24 month	No loss of correction over 95% graft integration, 65 non-ambulatory patients retrospective
Theologis et al. (2015) [18]	Allograft vs. autograft-AIC vs. BS	AIS	Posterior fusion + osteotomy	Radiologic fusion	24 month	No difference in fusion rates, BS group more pain, retrospective cohort analysis 3 groups 152AIC vs. 199 allograft vs. 110 BS
Sinagra et al. (2023) [19]	Allograft ± autograft	AIS + proximal junction kyphosis	Posterior fusion	Fusion, Cobb maintenance	24 month	88% (allograft) vs. 96% (combined), retrospective study of 78 patients
Bess et al. [20]	rhBMP-2	Adult spinal deformity	Posterior lumbar fusion	Fusion rate, complications	28 month	High fusion; 15% ectopic ossification Multicenter prospective analysis rhBMP-2 vs. non rhBMP-2 populations
Kim et al. (2016) [21]	rhBMP-2 vs. ICBG	Severe scoliosis (VCR)	PLSF or Combined Fusion	Fusion	4–14 year	Higher Fusion in rhBMP-2 group, matched cohort comparison 31 BMP patients vs. 32 ICBG patients
Gressot et al. (2012) [22]	rhBMP-2	Neuromuscular scoliosis	Posterior fusion	L5-S1 arthrodesis	11–62 month	92% fusion and Cobb angle, Retrospective review 11 pediatric patients
Dietz et al. (2011) [23]	rhBMP-2 vs. ICBG	Adult scoliosis	MIS posterior fusion	Healing time, pseudarthrosis	24 month	Only 1.14%pseusarthrosis rate in BMP population, retrospective cohort study rhBMP-2 vs. non rh-BMP-2
Giorgi et al. (2017) [24]	Collage-HA bone graft-RegenOss	Adult scoliosis	posterior fusion	Radiologic healing, implant stability	36 month	Union in 95% of cases, retrospective analysis 41 patients
Lerner & Liljenqvist (2013) [25]	Si-CaP + BMA	AIS	Posterolateral fusion	Fusion, Cobb loss, SRS-22, VAS	24 month	100% fusion; ≤7° loss; improved quality of life, prospective clinical study 21 patients
Ploumis et al. (2010) [26]	HA/collagen composite + BMA vs. cancellous allograft und local autograft	Lumbar Degenerative Scoliosis	Posterior fusion + decompression	Fusion, complications	24 month	Slower fusion to allograft+ autograft, same clinical outcomes 12 patients HA/collagen composite vs. 16 allograft
Lerner et al. (2009) [27]	β-TCP+ local bone vs. ICBG+ local bone	AIS	Posterolateral fusion	Fusion, correction loss, pain	24 month	100% fusion; less donor-site pain vs. ICBG, prospective randomized pilot study
Arnold et al. (2016) [28]	P-15 (i-Factor™) peptide vs. autograft	Adult ACDF	Anterior cervical fusion	Fusion, NDI, complications	12 month	88.97 vs. 85.82%; non-inferior outcomes, multicenter RCT 319 patients
Sardar et al. (2015) [29]	B2A-peptide ceramic-Prefix vs. ICBG	Adult degenerative deformity	Lumbar fusion	Fusion, ODI	12 month	100% high-dose vs. 78%; similar safety, multicenter RCT 24 patients 3 groups 9 ICBG vs. 8 Prefix 150 vs. 7 Prefix 750
Courvoisier et al. (2023) [30]	45S5 bioactive glass putty	AIS & non-idiopathic scoliosis in paediatric cohort	Posterior fusion	Fusion, Cobb loss, complications	24 month	100% fusion; <10° loss; no implant issues, retrospective study 43 patients
Passias et al. (2025) [31]	Novel integrative bone matrix-IBM	Adult multilevel deformity	Posterior fusion (1–15 levels)	Fusion, PROMs, AEs	12 month	100% fusion; improved function; no graft issues, retrospective analysis 20 patients
Animal Studies						
Zhang et al. (2020) [32]	Porous Ti cage + simvastatin hydrogel	Rhesus macaque lumbar fusion	Interbody fusion model	Bone ingrowth, spinal fusion	4 month	Enhanced bone ingrowth & fusion vs. controls, Controlled study with 6 macaques
Liang et al. (2025) [33]	3D porous tantalum cage	Sheep cervical & human PLIF	Interbody fusion	Osseointegration, clinical outcomes	12 month	Excellent osseointegration; favorable outcomes, Controlled study with 12 sheep and pilot study with 8 patients

**Table 2:** Summary of Grafts.

Graft Type	Source	Properties	Advantages	Limitations	Common Use Case
Autograft	Iliac crest (patient-derived)	Osteogenic, osteoinductive, osteoconductive	High fusion rates; biological compatibility	Donor-site morbidity; limited availability	Gold standard for short-segment fusions
Allograft	Cadaveric donor	Primarily osteoconductive	No donor-site morbidity; readily available	Slower incorporation; potential immunogenicity	Augmenting other grafts
Demineralized Bone Matrix (DBM)	Processed allograft tissue	Osteoinductive (variable)	Increases graft volume and biologic potential	Quality variability; cost factors	Extender/adjunct in long-segment fusion
Ceramics (HA, β-TCP)	Synthetic calcium-based	Osteoconductive	Biocompatible; resorbable	Brittle; limited mechanical strength alone	Often paired with biologics in pediatric fusion
Bioactive Glass	Synthetic silica-based	Osteoinductive via ionic release	Enhances angiogenesis; non-toxic degradation	Limited scoliosis evidence; less clinical data	Adjunct to other graft modalities
Bioactive Peptides	Synthetic peptides coated on ceramic/allograft	Osteoinductive via signaling peptides	Promotes fusion; avoids growth-factor side effects	Limited long-term data; emerging technology	Adjunct in cervical/lumbar fusion procedures
Bone Morphogenetic Proteins (BMPs)	Recombinant growth factors	Highly osteoinductive	Powerful bone formation	Costly; risk of inflammation/heterotopic bone	In complex deformity and revision cases
3D-Printed Grafts	Custom synthetic scaffold designs	Tunable porosity; design-specific precision	Anatomical customization; optimal scaffold architecture	Expensive; short-term data; experimental	Large/irregular defects; still under investigation

**Table 3:** Bias assessment of clinical studies.

Author(s) & Year [Citation]	Clear Inclusion Criteria	Standardized Measurement and Valid Methodology	Consecutive or Complete Inclusion	Clear Reporting of Demographics, Clinical Information, Outcomes	Appropriate Statistics	Recommendation
Crawford et al. (2013) [11]	Yes	Yes	Consecutive	Yes	Yes	Include
Erdem et al. (2025) [12]	Yes	Yes	Consecutive	Yes	Yes	Include
Ohtori et al. (2016) [13]	Yes	Yes	Unclear	Yes	Yes	Include
Yataganbaba et al. (2021) [14]	Yes	Yes	Consecutive	Yes	Yes	Include
Yang et al. (2023) [15]	Yes	Yes	Complete	Yes	Yes	Include
Lansford et al. (2013) [16]	Yes	Yes	Unclear	Yes	Yes	Include
Bui et al. (2014) [17]	Yes	Yes	Complete	Yes	Yes	Include
Theologis et al. (2015) [18]	Yes	Yes	Consecutive	Yes	Yes	Include
Sinagra et al. (2023) [19]	Yes	Yes	Complete	Yes	Yes	Include
Bess et al. [20]	Yes	Yes	Complete	Yes	Yes	Include
Kim et al. (2016) [21]	Yes	Yes	Complete	Yes	Yes	Include
Gressot et al. (2012) [22]	Yes	Yes	Complete	Yes	Yes	Include
Dietz et al. (2011) [23]	Yes	Yes	Complete	Yes	Yes	Include
Giorgi et al. (2017) [24]	Yes	Yes	Complete	Yes	Yes	Include
Lerner & Liljenqvist (2013) [25]	Yes	Yes	Complete	Yes	Yes	Include
Ploumis et al. (2010) [26]	Yes	Yes	Complete	Yes	Yes	Include
Lerner et al. (2009) [27]	Yes	Yes	Consecutive	Yes	Yes	Include
Arnold et al. (2016) [28]	Yes	Yes	Complete	Yes	Yes	Include
Sardar et al. (2015) [29]	Yes	Yes	Complete	Yes	Yes	Include
Courvoisier et al. (2023) [30]	Yes	Yes	Consecutive	Yes	Yes	Include
Passias et al. (2025) [31]	Yes	Yes	Consecutive	Yes	Yes	Include

## Data Availability

No new data were created or analyzed in this study.
